# Common Brain Structure Findings Across Children with Varied Reading Disability Profiles

**DOI:** 10.1038/s41598-017-05691-5

**Published:** 2017-07-20

**Authors:** Mark A. Eckert, Kenneth I. Vaden, Amanda B. Maxwell, Stephanie L. Cute, Mulugeta Gebregziabher, Virginia W. Berninger, Dyslexia Data Consortium, C. Beaulieu, X. V. Castellanos, C. Chiarello, T. Conway, L. Cutting, G. Dehaene-Lambertz, G. Eden, R. Frye, D. Giaschi, J. Gilger, F. Hoeft, M. Kibby, K. van Kriegstein, M. Kronbichler, C. M. Leonard, M. Milham, T. Odegard, R. Poldrack, K. Pugh, T. Richards, N. Rollins, K. Schneider, J. Talcott, B. Wandell

**Affiliations:** 1 Department of Otolaryngology – Head and Neck Surgery, Medical University of South Carolina, Charleston, SC 29425 USA; 2 Kennedy Krieger Institute, Baltimore, MD 21205 USA; 3 Department of Public Health Sciences, Medical University of South Carolina, Charleston, SC 29425 USA; 4 Department of Educational Psychology, University of Washington, Seattle, WA 98105 USA; 5 Department of Bioengineering, University of Alberta, Edmonton, Canada; 6 Department of Child and Adolescent Psychiatry, New York University, New York, USA; 7 Department of Psychology, University of California – Riverside, Riverside, USA; 8 The Morris Center, Gainesville, USA; 9 Peabody College of Education and Human Development, Vanderbilt University, Nashville, USA; 10 Department of Human Cognitive Development, INSERM, Paris, France; 11 Department of Pediatrics, Georgetown University, District of Columbia, USA; 12 Department of Neurology, Arkansas Children’s Hospital, Little Rock, USA; 13 Department of Ophthalmology and Visual Sciences, University of British Columbia, Vancouver, Canada; 14 Department of Psychological Sciences, University of California – Merced, Merced, USA; 15 Department of Psychiatry, University of California - San Francisco, San Francisco, USA; 16 Department of Psychology, Southern Illinois University, Carbondale, USA; 17 Human and Cognitive Brain Sciences, Max Planck Institute, Leipzeg, Germany; 18 Department of Neurology, University of Salzburg, Salzburg, Austria; 19 Department of Neuroscience, University of Florida, Gainesville, USA; 20 Department of Psychology, Middle Tennessee University, Murfreesboro, USA; 21 Department of Psychology, Stanford University, Stanford, USA; 22 Haskins Laboratories, New Haven, USA; 23 Department of Radiology, University of Washington, Seattle, USA; 24 Department of Radiology, University of Texas – Southwestern, Dallas, USA; 25 Department of Psychology, University of Delaware, Newark, USA; 26 Department of Psychology, Aston University, Birmingham, England

## Abstract

Dyslexia is a developmental disorder in reading that exhibits varied patterns of expression across children. Here we examined the degree to which different kinds of reading disabilities (defined as profiles or patterns of reading problems) contribute to brain morphology results in Jacobian determinant images that represent local brain shape and volume. A matched-pair brain morphometry approach was used to control for confounding from brain size and research site effects in this retrospective multi-site study of 134 children from eight different research sites. Parietal operculum, corona radiata, and internal capsule differences between cases and controls were consistently observed across children with evidence of classic dyslexia, specific comprehension deficit, and language learning disability. Thus, there can be common brain morphology findings across children with quite varied reading disability profiles that we hypothesize compound the developmental difficulties of children with unique reading disability profiles and reasons for their reading disability.

## Introduction

Dyslexia is a developmental reading disability that affects 5–15% of the U.S. population^1–3^. Children with reading disability can exhibit varied patterns of oral and written language problems^4, 5^ that include: (1) a classic dyslexia pattern with word-level decoding and spelling problems^6^; (2) comprehension-specific reading disability^7–9^; (3) and reading disability that occurs with specific language impairment or language learning disability^10, 11^. Neurobiologic studies of reading disability typically focus on one of these three behavioral profiles. In practice, children with quite varied language profiles based on clinical measures are included in reading disability samples^12^, which raises questions about the degree to which a particular reading disability profile (pattern of language skills) contributes to dyslexia findings.

Different reading disability profiles have been mapped to different neurobiological features^13–16^. This neurobiologic heterogeneity can limit effect sizes or produce inconsistent effects across studies when samples include multiple reading disability profiles, particularly when sample sizes are small. Thus, it is unclear as to whether there are common neurobiologic findings across children with reading disability or whether findings are due to a specific reading disability profile.

This behavioral heterogeneity problem is compounded by effects due to brain size when testing hypotheses about effects in locally specific brain regions. For example, low brain size is characteristic of children with oral and written language impairments^15^ and low brain size appears to occur with lower superior temporal sulcus gray matter volume in children with reading disability compared to controls^12^. Variance in brain size can dominate voxel-based effects in multi-site samples^12^ because brain size strongly covaries with voxel-based measures^17^.

Here we performed a case-control analysis of a multi-site dyslexia dataset to deal with brain size (total gray and white matter volume) and research site variance that can confound voxel-based effects. Parietal operculum and subcortical morphology differences were observed across reading disability profiles using Jacobian determinant images that represent the volumetric displacement of images to a brain template.

## Results and Discussion

### Matched-Pair Brain Morphometry Effects

**Figure 1:**
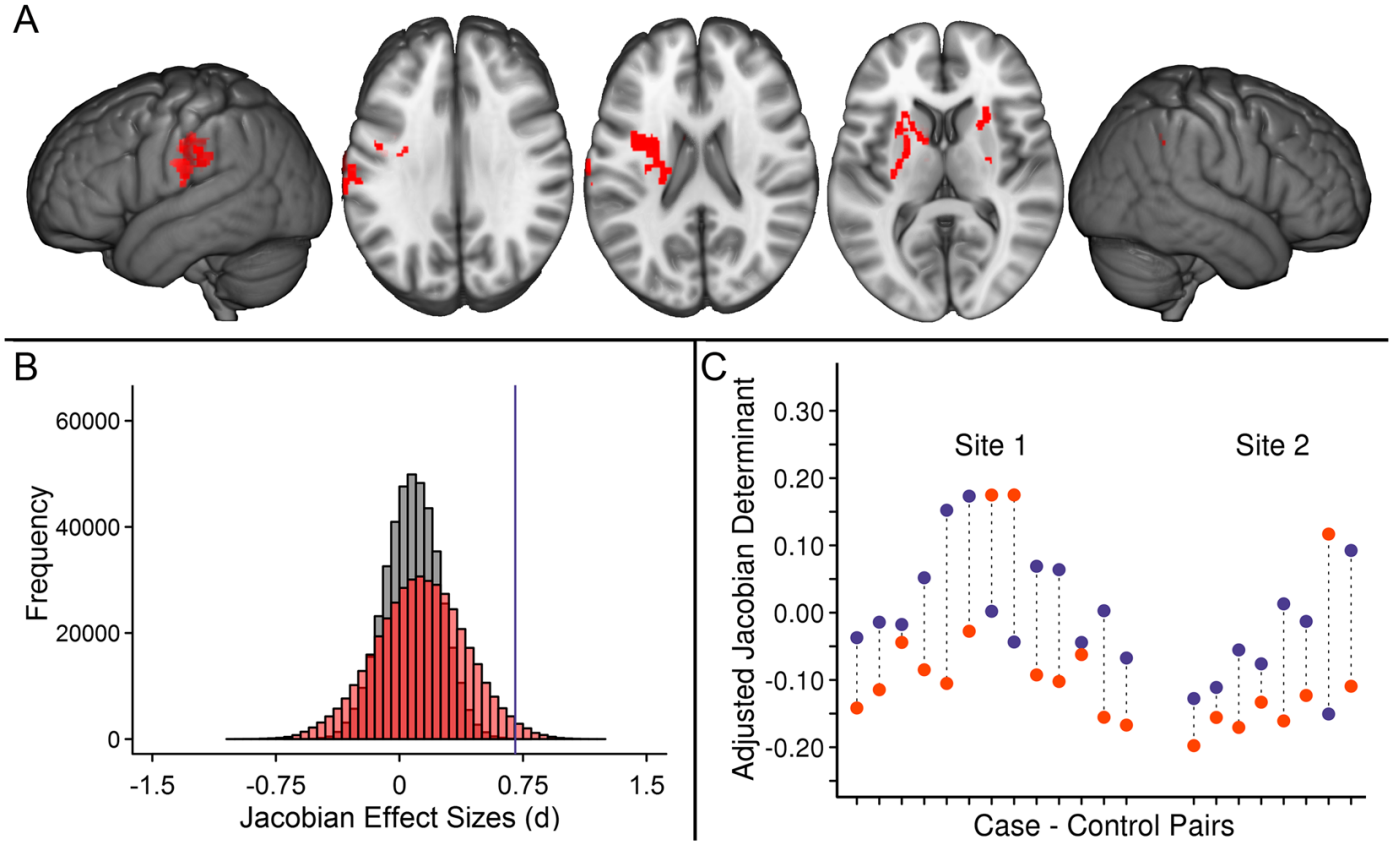
(**A**) Children with reading disability had brain images that required expansion to fit to a study-specific template compared to brain size and research site matched controls from the Dyslexia Data Consortium multi-site dataset (www.dyslexiadata.org). Parietal cortex, corona radiata, and internal capsule exhibited case-control differences were observed, in particular. Non-parametric permutation testing was used to correct for multiple comparisons (*p* < 0.05, red). (**B**) Histograms show the larger Cohen’s *d* effect sizes for the case-control design (red) compared to a typical unpaired group difference design (gray) across the 480,730 voxels within an inclusive gray and white matter mask. (**C**) The reason for statistical benefit of the case-control design is demonstrated by the relatively consistent left SII/anterior supramarginal gyrus (shown in **A**) differences in cases (orange) compared to matched controls (blue), despite the high morphologic variance within and across sites. Adjusted Jacobian determinant refers to the Jacobian or volumetric displacement values after correcting for brain size and research site-based propensity scores^58^.

Case-control pairs were optimally matched for sex and research site and to minimize case-control differences in age, total gray and white matter volume. One-sample t-tests of case-control Jacobian determinant difference images demonstrated that reading disability cases required more expansion of voxels to fit to a study-specific template (permutation-corrected *p* < 0.05; Table 1; Fig. 1A), particularly in the left parietal operculum and inferior parietal lobule [SII and supramarginal gyrus (SII/SMG)], as well as the bilateral corona radiata, and internal capsule (CR/IC) compared to controls (n = 67 pairs from 8 research sites; Supplementary Tables 1 and 2). The subcortical effects were spatially more extensive in the left hemisphere and extended from white matter underlying the pre-central gyrus, through the corona radiata, into the internal capsule, and down to the cerebral peduncle.

**Table 1:** Summary of the matched-pair brain morphometry results (*p* < 0.05 multiple comparison corrected) and contribution from each reading disability profile.

Brain Region	Voxel Cluster Size	Peak Coordinate	One-sample t-score N = 67/67	Poor Decoders t-score N = 33/33	Poor Comprehenders t-score N = 22/22	Generally Poor Readers t-score N = 12/12
L Inf. Parietal Lobule (SII/SMG)	458	−61, −17, 31	4.78***	2.50*	4.00***	2.09*
Left Precentral Gyrus, Corona Radiata, Internal Capsule	1409	−25, 3, 14	4.44***	2.75**	2.10*	3.11**
Right Corona Radiata, Internal Capsule	214	25, 21, 8	4.54**	2.68*	2.50*	2.81*

*p < 0.05, **p < 0.01, ***p < 0.001; t-scores are for the average difference in Jacobian determinant across each cluster shown in Fig. 1. N = #/# indicates the number of cases and controls in each analysis. These Jacobian determinant effects were not due to case-control differences in Perceptual Reasoning. For example, the association between case-control differences in the left motor region of interest and Perceptual Reasoning differences was *r*_(65)_ = 0.04, ns.

There were no significant voxel-based differences in volumetric displacement when performing unpaired group comparisons with permutation correction. Figure 1B presents the distribution of Cohen’s *d* effect sizes across the entire gray and white matter analysis space to show the increased sensitivity of the case-control design compared to an unpaired group design. Figure 1C shows that increased efficiency can occur because of variance within cases or controls and across research sites.

**Figure 2:**
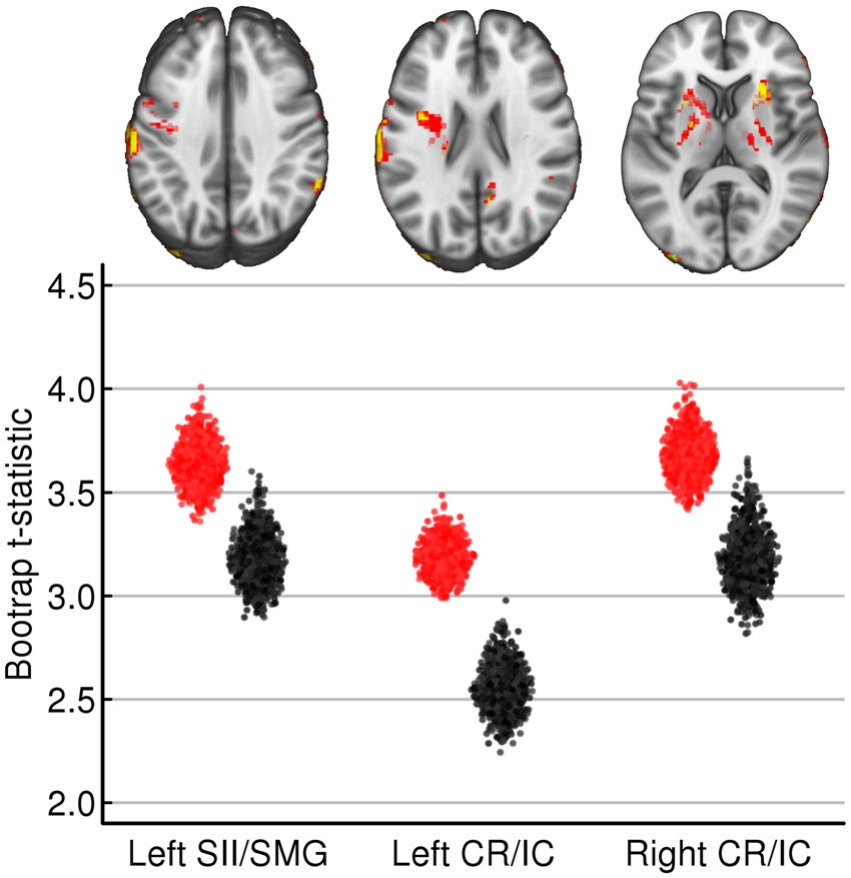
Well-matched and randomly matched case-control differences in Jacobian determinant/volumetric displacement. The top row of images shows the average t-score (thresholded with *t* ≥ 2.87 or the minimum t-score within the permutation corrected results in Fig. 1) across 1000 well matched (red clusters) and randomly matched (yellow clusters) case-control samples. Note that the extent of the effects are consistently smaller when a reading disability case was randomly matched with a control compared to when cases and controls were well-matched for brain volume and research site, even though the same 134 participants were examined in each set of analyses. The scatterplots show the distribution of 1000 t-scores pooled within the significant left SMG, Left CR/IC, and Right CR/IC clusters from Fig. 1. Note the consistently higher average t-score for the well-matched case-control samples (red) compared to the randomly matched case-control samples (gray).

Bootstrap resampling was used to examine how the case-control matching affected the Jacobian determinant results. Case-control matches were resampled without replacement to form 1000 well-matched pairs (paired within stratified brain size and research site groups) and 1000 randomly matched pairs (paired across brain size and research sites). We then performed t-tests to establish the distribution of case-control differences. Because the same 134 individuals were used to form both bootstrap statistic distributions, differences in the voxel-level results were reflective of the quality of case-control matches. While the well-matched samples yielded results that were similar to the optimally matched results show in Fig. 1A, these effects were diminished in spatial extent and magnitude for the randomly paired case-control samples (Fig. 2). The relatively smaller volumetric displacement differences when cases and controls were not matched across research sites are consistent with the Fig. 1C demonstration of how between site variation can negatively impact a result.

### Reading Disability Profile Effects

**Figure 3:**
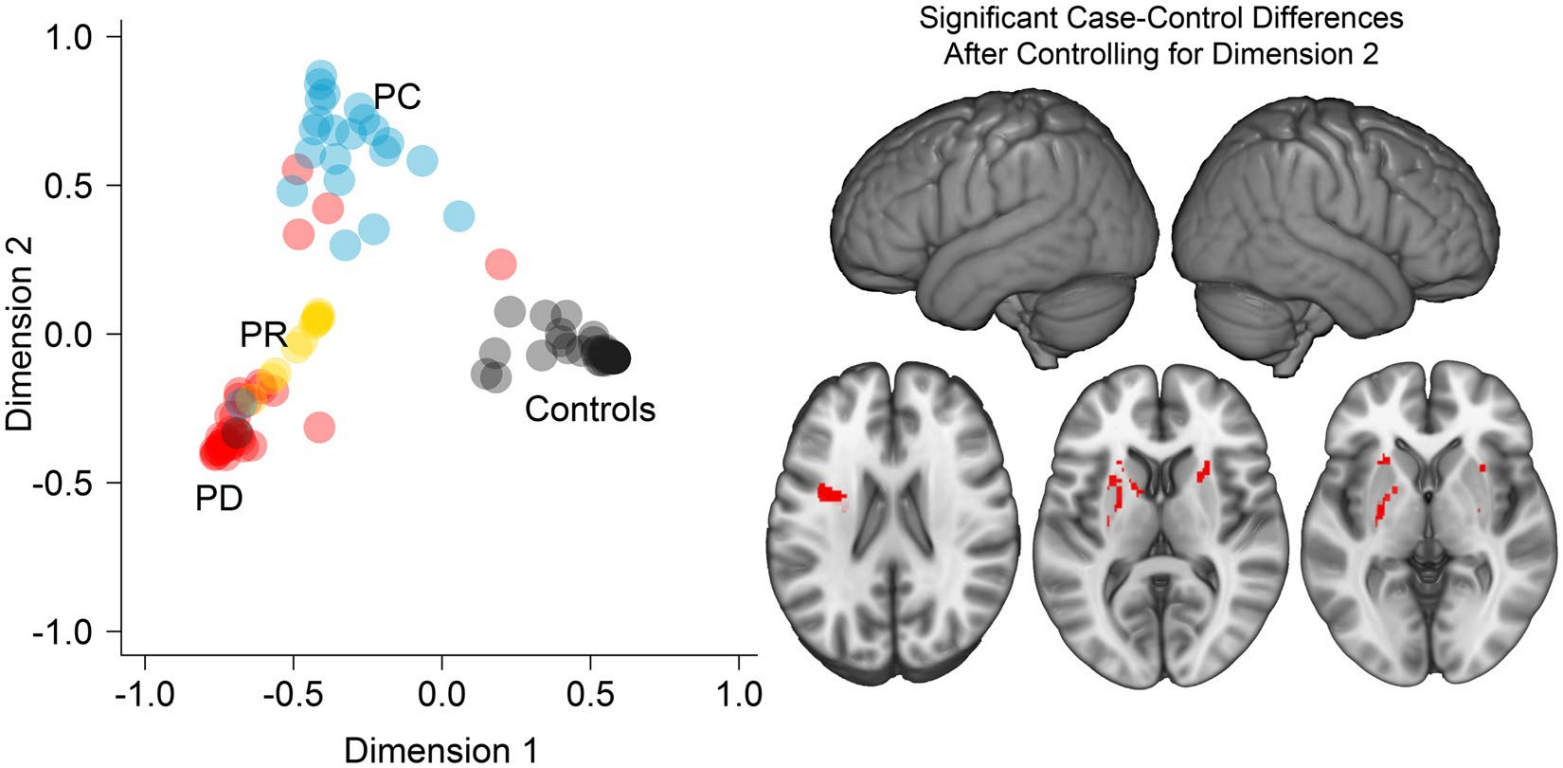
Multidimensional scaling demonstrates the extent to which reading disability profiles differ from controls. Left plot: Across participants, a larger Dimension 1 value was significantly associated with higher Verbal Comprehension, Word Attack, Word Identification, Passage Comprehension, and Rapid Naming performance (*p*s < 0.001). Dimension 2 was negatively associated with Verbal Comprehension (*r*_(132)_ = −0.21, p = 0.02) and negatively associated with Passage Comprehension (*r*_(132)_ = −0.17, p = 0.05). The color of the symbols refers to the expert-rater classification (PD: Poor Decoder; PR: Generally Poor Reader; PC: Poor Comprehender) and therefore shows which participants were inconsistently classified by the Random Forest algorithm. Right brain images: The subcortical effects, but not the left SII/SMG effect, shown in Fig. 1 remain significant after controlling for matched-pair differences﻿ in Dimension 2. This result is consistent with Table 1 showing that Poor Comprehenders, which largely affect Dimension 2, exhibited pronounced left SII/SMG differences between cases and controls. There were not significant effects after controlling for dimension 1.

Clinician expert-raters classified the reading disability cases according to the three reading disability profiles described above based on evidence of: (1) poor word and non-word phonological decoding (Poor Decoders); (2) relatively specific reading comprehension problems (Poor Comprehenders); and (3) relatively poor decoding, reading comprehension, and verbal comprehension [Generally Poor Readers; (Supplementary Tables 2 and 3 for behavioral profiles and classification criteria)]. These profiles were defined based on the extant literature on reading disability profiles^4, 5^ and the raters’ considerable clinical experience assessing and treating children with reading disability. Random Forest was then used to show that these profiles could be consistently classified as different from controls (94% accuracy) using a machine learning algorithm. These unique patterns (profiles) of reading disability are shown in Fig. 3, where multidimensional scaling was used to examine the distinctiveness of the reading disability profiles and controls. Analysis of variance demonstrated significant differences in multi-dimensional space between the reading disability profiles (Dimension 1: *F*_(2,66)_ = 27.02, p < 0.001; Dimension 2: *F*_(2,66)_ = 90.13, p < 0.001). Post-hoc comparisons revealed that all three reading disability profiles exhibited significant differences between each other for both dimensions (*p*s = 0.01 to *p* < 0.001).

The multidimensional behavioral space provided a quantitative metric that characterized variance within and between the reading disability profiles, as well as how different each reading disability case was from their matched-pair control. We examined the extent to which the case-control differences in multidimensional space were associated with case-control differences in Jacobian determinant values. There were no significant effects for either dimension. However, including these behavioral space variables in the voxel-based analyses resulted in non-significant voxel-based effects across the sample when Dimension 1 case-control differences were controlled. In addition, the left SII/SMG region was no longer significant when controlling for Dimension 2 case-control differences in a separate model. This was due to a modest reduction in effect size after controlling for the dimension that largely differentiated Poor Comprehenders from the other reading disability profiles and controls. The subcortical white matter effects were relatively resilient to controlling for case-control differences in behavioral Dimension 2 (Fig. 3). Thus, case-control differences in subcortical morphology appeared to be more likely when there were large case-control differences across reading measures.

Corona radiata findings have been reported in diffusion imaging studies of dyslexia^18–20^ and there have been some questions about the extent to which these effects explain or are additive to arcuate fasciculus findings^21, 22^. While it is unclear if the parietal operculum results in this study reflect atypical arcuate fasciculus white matter, it is worth noting that there was no significant relation between the left SII/SMG and left CR/IC Jacobian determinant differences across reading disability profiles (*r*_(65)_ = 0.13, ns). This observation is consistent with the results of controlling for Dimension 2 on the left SII/SMG case-control differences, but not the left CR/IC differences.

### Parietal Operculum/SMG Findings Interpretation

The left parietal operculum has been observed to exhibit atypical activation in functional imaging studies of dyslexia^23, 24^. Left secondary somatosensory cortex (SII), in particular, exhibits increased responsiveness when people attend to speech^25^. A similar parietal operculum region (SII to SMG) is thought to support phonological processing by providing a working memory mechanism to “clean up” noisy phonological representations^26^. These findings appear to be consistent with evidence that this region supports mapping orthographic to phonologic representations^27^. They also suggest that atypical orthographic development would limit the benefit from orthographic consistency on speech processing in children with reading disability, as demonstrated in refs 28 and 29.

A role for SII and SMG cortex in mapping orthographic to phonologic representations predicts that these regions are engaged when people read aloud^27^. SII is engaged during speech production tasks^30^ and also exhibits gray matter density changes that track with changes in Verbal IQ^31^. Longitudinal changes in left SII gray matter density appeared to predict changes in Verbal IQ because of reading experience^31^. In support of this experience explanation, parietal cortex morphology (Jacobian determinant) was related to individual differences in reading ability for ~8.4 year old children, but not in the same children when the data were collected a year earlier^32^. Moreover, adults acquiring literacy late in life had increased SMG gray matter volume compared to illiterate adults^33^. The parietal operculum effects in the current study were observed across reading disability cases from 7.33–12.67 years of age, but the differences appeared to be most pronounced among the younger children (Parietal operculum/SMG Jacobian difference * reading disability case age: *r*_(65)_ = −0.32, p = 0.008).

One way parietal cortex could “clean up” noisy representations is through accumulating evidence to aid in perceptual decision-making^34^. Children with reading disability may not be able to make efficient use of working memory to accumulate sensory evidence for mapping orthographic and phonologic information (Poor Readers and Poor Decoders) or for following the meaning of connected speech or text (Poor Readers and Poor Comprehenders). Working memory measures may have demonstrated impaired phonological working memory across all three reading disability profiles^35–38^.

### Subcortical Findings Interpretation

The corona radiata and internal capsule differences appear to be consistent with previous subcortical white matter and gray matter results^39–41^, which again may relate to diffusion imaging findings linking the CR/IC to reading development^18, 40^. Given that the CR/IC effects were particularly evident in the Generally Poor Readers (Table 1) who can have a broad range of behavioral impairments, there is a possibility that these effects reflect perinatal events that affect periventricular white matter development^42^ and result in reading disability^43, 44^. The results suggest that our multi-site dataset included children who experience motor and perhaps somatosensory impairments that contribute to their risk for reading disability, particularly when there is language impairment^45^. These potential motor and somatosensory effects appear to be independent of spatial impairments as there was no evidence that the subcortical effects were due to case-control differences in Perceptual Reasoning.

### Summary

Matched-pair brain morphometry demonstrated significant differences in brain morphology between reading disability cases and controls. The parietal operculum appears to be a target for tracking the effectiveness of interventions^46^, and may serve as an early marker of reading disability, if present in at-risk children before formal reading instruction^47, 48^. In contrast, the subcortical white matter findings within putative descending fiber tracts could be consistent with a procedural learning deficit hypothesis for language disabilities^49^ and perhaps a cerebellar deficit hypothesis for dyslexia^50^. We were unable to test these hypotheses because working memory, motor, and somatosensory data were not available in our retrospective dataset. However, investigators can address these hypotheses in prospective studies. Together, these results indicate that there can be common brain morphology findings across children with quite varied reading disability profiles. We hypothesize that atypical development in SMG and CR/IC regions compound reading difficulties in children with unique reading disability profiles that arise from unique neural underpinnings.

## Methods

### Participants and Behavioral Data

The data included in this study were collected as part of a project to develop methods for retrospective analysis of multi-site data and are part of a Dyslexia Data Consortium database (www.dyslexiadata.org). These existing data were collected from contributing sites where the human subject review boards provided approval to share de-identified data that were collected under the appropriate human subjects guidelines of those institutions and with informed consent. Data de-identification protected the privacy of subjects in accordance with HIPAA regulations^51^. Reception of these “non-human” de-identified data was approved by the Medical University of South Carolina (MUSC) Institutional Review Board (IRB) and the research described here was performed in accordance to MUSC IRB regulations and guidelines.

Data from 247 children (41% female; age range = 9.65, sd = 1.63) were selected from the multi-site dataset based on having T1-weighted images and standardized scores from the: (1) Woodcock-Johnson IIIR or Woodcock Reading Mastery Tests (Word Attack, Word Identification, and Passage Comprehension)^52, 53^; (2) the Wechsler Intelligence Scales for Children or the Wechsler Abbreviated Scales of Intelligence (Verbal Comprehension and Perceptual Reasoning)^54, 55^; and (3) the Comprehensive Test of Phonological Processing Rapid Automatized Naming or the Rapid Alternating Stimulus Tests^56, 57^. A subset of these cases (n = 134; 43% female; mean age = 9.61, sd = 1.57) was included in brain morphology analyses after identifying (1) cases who could be consistently classified as having unique reading disability profiles (39 inconsistently classified by expert raters) and (2) cases and controls that could be matched according to brain size and research site (57 without matches). Controls were included if they had Word Attack, Word Identification, and Passage Comprehension scores above the 25^th^ percentile, while reading disability cases were selected if they scored below the 25^th^ percentile for one of these measures.

### Reading Disability Profile Classification

Two clinically certified Speech-Language Pathologists with considerable experience assessing children with reading disabilities examined the behavioral data to classify reading disability cases into classic dyslexia (Poor Decoders), comprehension-specific (Poor Comprehenders), and multiple language impairment profiles (Generally Poor Readers). Guided by the empirical literature^4–11, 58^ and input from a Psychologist with extensive clinical experience with specific learning disabilities (SLDs) and 25 years of experience directing NIH-supported research on SLDs, *Poor Decoders were classified based on*: (1) Verbal Comprehension standard scores that were within the average to above-average range or above the 25^th^ percentile; (2) Word Attack standard scores that were typically below the 25^th^ percentile; (3) Word Identification and Passage Comprehension standard scores that were typically consistent with Word Attack scores; and (4) RAN standard scores that were typically below the 25^th^ percentile. *Poor Comprehenders were classified based on*: (1) Verbal Comprehension scores that met the same criteria as Poor Decoders; (2) Word Attack scores that were above the 25^th^ percentile; (3) Word Identification and Passage Comprehension scores that were typically at or below the 25^th^ percentile; (4) RAN scores that were typically greater than the 16^th^ percentile; and (5) children were typically older than 7–8 years to consider the effects of developmental and academic exposure on comprehension. *Generally Poor Readers were classified based on* Verbal Comprehension, Word Attack, Word Identification, and Passage Comprehension scores that were at or below the 25^th^ percentile. Supplemental Table 2 presents the means for these variables by the expert-rater profiles and their matched controls. Expressive and receptive language and executive function measures were not available to further confirm the Poor Reader and Poor Comprehender classifications, respectively.

Supplementary Table 3 presents the classification rules above that were established based on the reading disabilities literature. The two expert raters met to discuss the classification rules prior to classifying the cases. Across 144 reading disability cases from the original sample of 247 reading disability and control participants, intra-rater reliability for the primary rater (Chronbach’s alpha = 0.94) was expectedly higher than inter-rater reliability (Chronbach’s alpha = 0.79).

A Random Forest classifier^59^ was developed using the consistently classified reading disability cases to demonstrate that the clinical expert classified profiles could be distinguished from each other and from controls based on their behavioral profiles using an automated approach. The analyses were carried out using the R statistics package (R version 3.2.3 with packages: caret 6.0–64, randomForest 4.6-12). The Random Forest was tuned (*mtry* parameter = 2; number of trees = 500) with a five step parameter search, then classified groups within the training dataset based on a leave-one-out cross validation test (LOOCV). The LOOCV test iteratively trains the Random Forest algorithm using all but one case from the training dataset, then classifies the remaining case to approximate independent train and test data. Classification accuracy was determined by whether the LOOCV classification of each case matched its expert-based label.

Random Forest predicted the expert-rater classifications of Poor Decoders, Poor Comprehenders, Generally Poor Readers, and controls with 94% accuracy using the same behavioral and age variables that were used by the expert-raters. Each variable, perhaps with the exception of age, contributed to classification accuracy (decrease in accuracy with exclusion of the following variable: Word Attack = 48.21%; Passage Comprehension = 39.27%; Verbal Comprehension = 29.66%; Word Identification = 26.16%; RAN = 14.39%; Age = −0.43%). Random Forest misclassified cases were likely to have a Verbal Comprehension or Word Attack score, for example, at a boundary that distinguished the reading disability profiles. These results (1) provide insight into the behavioral variables that were most important for reading disability profile classification and (2) demonstrate that there were unique behavioral profiles in the dataset. As described below, the Random Forest classification probabilities for each reading disability profile and controls were used to perform multidimensional scaling for the 134 cases and controls. This analysis generated two spatial dimensions to confirm that the profiles were clearly different from each other and from controls (Fig. 3).

### Imaging Data and Pre-Processing

Bias-field corrected and Rician denoised^60^ T1-weighted images from the 8 different research sites (Supplemental Table 1) were rigidly aligned to the MNI T1 template using the SPM12 Coregister function. Gray matter, white matter, and CSF probability in each voxel were estimated using the SPM12 Segment function. The resulting native space gray and white matter images were used to obtain an overall sum of the gray or white matter probabilities as an estimate of total gray or white matter volume. The native space segmented images gray matter, white matter, and CSF images were spatially transformed into a study-specific anatomical space using the SPM12 diffeomorphic (DARTEL) normalization procedure^61^. Jacobian determinant images were calculated from the normalization parameters to create images in template space that represented the amount of volumetric displacement that was required to fit an image to the template. These image pre-processing methods and the SPM default parameters were selected to be aligned with commonly used voxel-based morphometry protocols to facilitate replication^62^.

### Statistics

#### Pair Matching

Case-control pairs were identified by (1) creating strata of cases and controls according to smaller or larger brain size (total volume of the gray and white matter segmented images) and research site, and then (2) selecting unique pairs of optimally matched cases and controls within each stratum. Specifically, coarsened exact matching (CEM; R package: cem, version 1.1.17) was used to create the strata for 176 participants that included participants with smaller or larger total brain sizes who were from the same research site. Propensity scores were estimated for each subject with a logistic regression that predicted case-control status using research site and total brain size. A nearest-neighbor algorithm (R-package: MatchIt, version 2.4.21) was used to identify the most similar case-control pairs within each stratum on the basis of the propensity score, the match-quality weight from CEM, and total brain size. Because each case-control pair was required to include participants that did not occur in any other pair, the matching procedure reduced the sample size to 134 subjects (i.e., 67 pairs). Paired-sample t-tests were performed to determine that there were no significant differences in the resultant matched pairs with respect to age, total gray matter volume, total white matter volume, or total brain size (*p*s > 0.25; Supplemental Table 2). In addition, there was no significant difference in distribution of sex between the cases and controls (24 female cases, 43 male cases, 28 female controls, 39 male controls: *Chi*-*square* = 0.28, *p* = 0.59). There also was no significant difference in propensity score between the reading disability cases and controls (t_(132)_ = −0.33, ns) and no significant case-control differences in propensity scores between the reading disability profiles (F_(2,66)_ = 0.01, ns).

The propensity scores that were calculated for the selection of case-control matches were also used to adjust or residualize the 134 Jacobian determinant images for brain size and research site effects prior to creating the control-case difference images (R-package: AnalyzeFMRI, version 1.1.16). This approach further limited the impact of these potential nuisance variables on the results that can lead to bias amplification, particularly when their effect on a dependent variable is stronger than the predictor variable of interest^63^. The CEM procedures were chosen because we had observed that covarying or residualizing brain volume differences inflated case-control difference image effects when case and control matching was performed without considering brain size, which was confirmed with simulation analyses when a strongly confounded nuisance variable was residualized. Supplemental Fig. 1 shows how residualizing for a highly collinear nuisance variable can bias results and shows that the results of this study were less likely to be affected because we performed an initial selection of cases and controls to match for brain size and research site. As with any regression procedure, our observations suggest that (1) caution should be taken when adjusting for nuisance variables that have high collinearity with variables of interest and that (2) a case-control design is one solution for dealing with this problem.

#### Paired and Unpaired Comparisons

Statistical analyses were performed using (1) an unpaired group difference approach that is typically used in neuroimaging studies of group differences and (2) the matched-pair case-control approach. The results demonstrated a statistical advantage of the matched-pair approach over the unpaired approach, at least for a sample size of 134 subjects. The FSL randomise function was used to perform these voxel-based comparisons with 10,000 permutations to control for multiple comparisons. Threshold free cluster enhancement (TFCE) was used to optimize sensitivity to focal large effects and spatially broader smaller effects^64^ for the unsmoothed Jacobian determinant images that can represent shape and gross volumetric effects^17, 65^.

#### 1000 Well-matched and 1000 Random Bootstrap Samples

To ensure that case-control results were not dependent on the sampling of one set of case-control pairs, voxel-based t-test comparisons were performed for 1000 well-matched case-control samples to establish confidence intervals (Fig. 2) for the significant case-control differences (Fig. 1). This was repeated for 1000 randomly paired case-control samples to determine the extent to which the results would be affected when cases and controls are not carefully matched. The key difference between well-matched and randomly matched samples was that a cases were paired with controls across research sites for the randomly matched samples. Neither well-matched nor randomly matched samples exhibited significant case-control differences in total gray and white matter volume and age (all *p*s > 0.26).

#### Multi-dimensional Scaling and Profile Effects

Finally, multi-dimensional scaling was performed in R with the Random Forest classification probabilities using the cmdscale function. This procedure provided a Euclidian distance estimate for each participant relative to the others based on their reading disability profile classification probabilities. Two dimensions provided a goodness of fit = 0.87. Inspection of the Random Forest probabilities that suggested Poor Decoders and Generally Poor Readers fell along a continuum of Poor Decoder to Poor Reader, suggesting that a 3^rd^ dimension was not necessary to differentiate these profiles, as further demonstrated in Fig. 3 by the distribution of Poor Decoders to Generally Poor Readers. The 2 dimensions appeared to characterize the severity of reading skill impairments (phonological decoding and real word decoding) and the severity of comprehension impairment.

The multi-dimensional distance variables for each dimension were subtracted between a case and control to obtain case-control distance differences for each dimension. These two variables were used to determine the extent to which case-control brain structure differences could be attributed to the magnitude of behavioral difference between each case and control for metrics that were sensitive to the distribution of reading disability profiles. This provided a quantitative variable to test profile effects that considered the behavioral profile of both the case and control. Permutation tests (10,000) with FSL randomise (TFCE) were performed to relate difference images to one of the dimension difference variables at a time, to determine the extent to which the effects shown in Fig. 1 were dependent on the magnitude of case-control differences along reading skill and comprehension dimensions.

## Electronic supplementary material

Supplementary Information
