# Supplementary material for: Common Brain Structure Findings Across Children with Varied Reading Disability Profiles
Source: Sci Rep. 2017 Jul 20;7:6009. doi: 10.1038/s41598-017-05691-5 (PMC5519686; doi:10.1038/s41598-017-05691-5)
Supplement: Supplementary file 1 — Supplementary Information [file 41598_2017_5691_MOESM1_ESM.docx]

**Supplemental Materials for**

**Common Brain Structure Findings Across Children with**

**Varied Reading Disability Profiles**

**Mark A. Eckert** ^1^*****

**Kenneth I. Vaden, Jr.** ^1^

**Amanda B. Maxwell** ^1^

^1^ Department of Otolaryngology – Head and Neck Surgery

Medical University of South Carolina

**Stephanie L. Cute** ^2^

^2^Kennedy Krieger Institute

**Mulugeta Gebregziabher** ^3^

^3^ Department of Public Health Sciences

Medical University of South Carolina

**Virginia W. Berninger** ^4^

^4^ Department of Educational Psychology

University of Washington

**Dyslexia Data Consortium**

**Supplementary Table 1.** T1-weighted image parameters from the 8 study sites.

| Site | Manufacturer | Field  Strength  (T) | Image  Dimension  (mm) | Slice  Thickness  (mm) | TR  (msec) | TE  (msec) | Flip  Angle  (deg) |
| --- | --- | --- | --- | --- | --- | --- | --- |
| 1 | Siemens | 1.5 | 256 x 256 x 160 | 1.60 | 25.00 | 4.60 | 30 |
| 2 | Siemens | 3.0 | 176 x 240 x 256 | 0.90 | 2250.00 | 3.96 | 9 |
| 3 | Siemens | 3.0 | 128 x 256 x 256 | 1.33 | 6.60 | 2.90 | 8 |
| 4a | GE | 1.5 | 124 x 256 x 256 | 1.2 | 11.10 | 2.20 | 25 |
| 4b | GE | 1.5 | 124 x 256 x 256 | 1.40 | 11.10 | 2.20 | 25 |
|  |  |  |  |  |  |  |  |
| 5 | Siemens | 3.0 | 160x 256 x 256 | 1.00 | 1600.00 | 3.37 | 15 |
| 6 | Philips | 1.5 | 170 x 256 x 256 | 1.00 | 8.02 | 3.69 | 7 |
| 7 | GE | 1.5 | 181 x 217 x 181 | 1.00 | 6.00 | 63.00 | -- |
| 8 | Siemens | 1.5 | 160 x 256 x 256 | 1.00 | 2000 | 3.65 | 8 |

The imaging parameters above were obtained from de-identified DICOM information when available, but from related manuscripts or image header information, which is why flip angle information is missing for data from one site. The relatively long TRs above were used for inversion recovery acquisitions (inversion times: site 2, 900 ms and site 5, 640 ms). 4a,b- Data were obtained for 2 different studies from a research site.

**Supplementary Table 2.** Mean and standard deviations (SD) for the expert rater reading disability (RD) profiles and their matched control cases.

| **Profile** | **Demographic,** | **Reading Disability** | | **Control** | |  |  | |
| --- | --- | --- | --- | --- | --- | --- | --- | --- |
|  | **Reading,** |  |  |  |  |  |  |  |
|  | **Brain Size** |  |  |  |  |  |  |  |
|  | **Variables** | Mean | SD | Mean | SD | t-score p-value | | |
| Poor Decoders | Age | 9.7 | 1.64 | 9.56 | 1.67 | -0.44 | | ns |
|  | WA | 84.21 | 7.33 | 109.3 | 11.82 | 10.99 | | *** |
|  | WID | 81.64 | 8.33 | 107.97 | 10.31 | 11.45 | | *** |
|  | PC | 85.48 | 15.75 | 107.73 | 9.64 | 6.63 | | *** |
|  | RAN | 89.06 | 23.05 | 103.38 | 13.63 | 4.31 | | *** |
|  | VIQ | 104.15 | 9.62 | 118.15 | 11.61 | 5.05 | | *** |
|  | PIQ | 103.66 | 14.39 | 114.55 | 15.41 | 2.69 | | * |
|  | Total GM | 851.49 | 87.07 | 848.42 | 99.09 | -0.25 | | ns |
|  | Total WM | 393.91 | 47.43 | 409.1 | 54.88 | 1.69 | | ns |
|  | Total GM+WM | 1245.40 | 135.62 | 1257.51 | 123.50 | 0.68 | | ns |
| Poor Comprehenders | Age | 9.49 | 1.49 | 9.92 | 1.76 | 0.83 | | ns |
|  | WA | 98.36 | 7.89 | 110.14 | 13.97 | 3.18 | | ** |
|  | WID | 89.36 | 9.85 | 109.05 | 12.64 | 5.21 | | *** |
|  | PC | 85.23 | 3.9 | 108.32 | 11.33 | 7.84 | | *** |
|  | RAN | 91.98 | 7.55 | 101.25 | 10.85 | 3.17 | | ** |
|  | VIQ | 101.59 | 8.48 | 120.55 | 14.6 | 6.2 | | *** |
|  | PIQ | 103.55 | 14.37 | 113.73 | 10.74 | 2.36 | | * |
|  | Total GM | 862.09 | 98.29 | 870.86 | 86.41 | 0.67 | | ns |
|  | Total WM | 409.22 | 51.88 | 408.06 | 60.68 | -0.11 | | ns |
|  | Total GM+WM | 1271.30 | 133.94 | 1278.92 | 125.52 | 0.44 | | ns |
| Generally Poor Readers | Age | 9.14 | 1.31 | 9.57 | 0.97 | 1.06 | | ns |
|  | WA | 79.67 | 6.24 | 118 | 13.61 | 7.94 | | *** |
|  | WID | 72.92 | 8.65 | 116.33 | 12.35 | 10.3 | | *** |
|  | PC | 72.83 | 6.66 | 111.33 | 8.35 | 11.16 | | *** |
|  | RAN | 82.71 | 8.08 | 103.13 | 11.03 | 4.55 | | *** |
|  | VIQ | 82.5 | 5.35 | 114 | 12.75 | 10.13 | | *** |
|  | PIQ | 88.33 | 11.89 | 115.5 | 18.29 | 4.75 | | *** |
|  | Total GM | 832.92 | 82.25 | 846.02 | 60.59 | 0.9 | | ns |
|  | Total WM | 392.13 | 49.69 | 388.69 | 39.68 | -0.37 | | ns |
|  | Total GM+WM | 1225.05 | 127.79 | 1234.71 | 96.05 | 0.44 | | ns |

WA – Word Attack; WID – Word Identification; PC – Passage Comprehension; RAN – Rapid Automatized Naming; VIQ – Verbal Comprehension/IQ; PIQ – Perceptual Reasoning/IQ; Total GM – Total Gray Matter Volume; Total WM – Total White Matter Volume. ns – nonsignificant; * p < 0.05; *** p < 0.001. t-score is from a paired samples t-test of the cases and their matched controls. There were no significant differences in sex across profiles and controls (Chi-square = 0.28, p = 0.59): Poor Decoders: 11 females / 21 males. 1 had missing PIQ data; Poor Decoder Matched Controls: 12 females / 20 males; Poor Comprehenders: 11 females / 11 males; Poor Comprehender Matched Controls: 7 females / 15 males; Generally Poor Readers: 6 females / 7 males; Generally Poor Reader Matched Controls: 5 females / 8 males.

**Supplementary Table 3.** Expert rater reading disability profile classification guidelines.

|  | **Poor Decoder** | **Poor Comprehender** | **Generally Poor Reader** |
| --- | --- | --- | --- |
| **Verbal Comprehension**  *Caveat* | Typically > 25^th^ percentile | Typically > 25^th^ percentile | < 25^th^ percentile |
|  | *Low average range (scores of 85-89) for older children (>10 years) where failure to respond to instruction can affect performance* | *Low average range (scores of 85-89) when viewed in relation to higher Word Attack and lower Passage Comprehension scores* |  |
| **Word Attack** | Typically < 25^th^ percentile | > 25^th^ percentile | < 25^th^ percentile |
| **Word Identification** | Typically < 25^th^ percentile | Typically < 50^th^ percentile | < 25^th^ percentile |
| **Passage Comprehension**  *Caveat* | < 25^th^ percentile | < 25^th^ percentile | < 25^th^ percentile |
|  | *Could be > 25^th^ percentile if Verbal Comprehension was also in the average to above average range (scores > 100)* |  |  |
| **Rapid Automatized Naming**  *Caveat* | Any | Typically > 16^th^ percentile | Typically < 25^th^ percentile |
|  | *Scores < 25^th^ percentile confirmed a Poor Decoder classification* |  |  |

Across profiles, Verbal Comprehension was considered first in classifying cases and typically identified Generally Poor Readers. Word Attack was then examined to rule out Poor Comprehenders and identify possible Poor Decoders. Passage Comprehension was then examined to identify potential Poor Comprehenders who otherwise had test scores in the normal range. Word Identification was more variable across cases, but was helpful in differentiating Poor Comprehenders from the other profiles. Rapid Automatized Naming guided classification of Poor Decoders, but typically contributed less to classification after considering the other variables, which also is demonstrated in the Random Forest classification results described above. Average for the standardized scores is 100 with a 15-point standard deviation.

**Supplemental Figure 1**


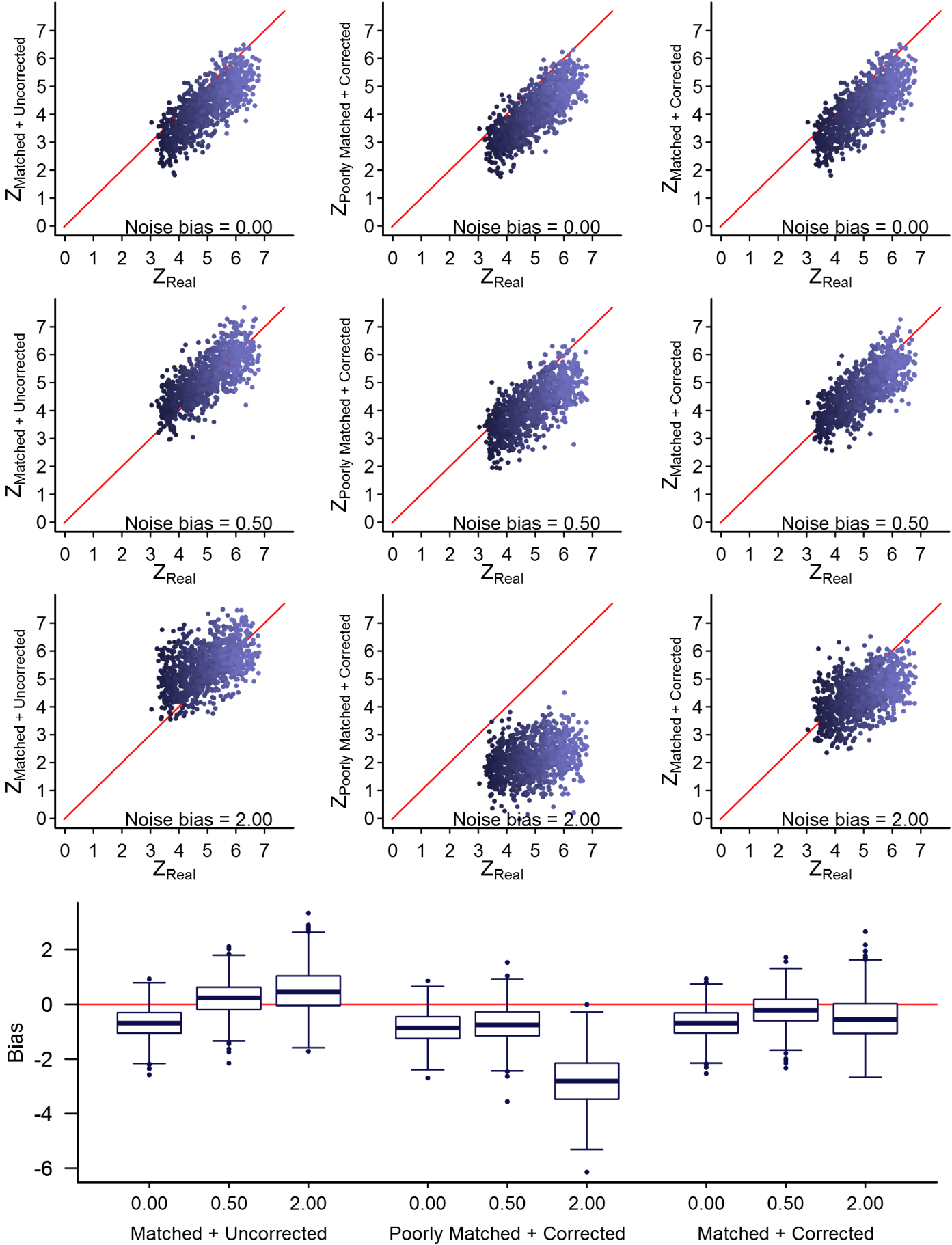


**Supplemental Figure 1.** A bootstrap simulation with 1000 pseudo-randomly generated gray matter vectors and a case-control difference that ranged between 0.5-1 standard deviations between 100 cases and 100 controls (d = 0.5 to 1). Each simulation included three noise manipulations, which were combined in the following percentages: 1) nine sites (40%), 2) varied brain size (40%), and 3) a latent noise variable (20%). A specified level of noise bias (0, 0.5, 2; scatterplot rows) was added to both the brain size noise and latent noise, only for controls, to simulate a difference in the noise that was confounded with the difference of interest. Distorted gray matter values were produced by adding the combined noise vectors to the gray matter vector to simulate confounding variance in observed data. The difference calculated across the entire sample of paired cases and controls in the simulated gray matter values without noise was used as the ground truth (Z_Real_) for estimating biases in paired and/or adjusted data. Similar to pair selection in the real data, matched pairs were created by stratifying the simulated data within each site according to small or large brain size, followed by nearest neighbor matching that used simulant’s propensity score and brain size. Poorly matched pairs were also generated by selecting nearest neighbors for cases with controls based on the inverse of their propensity scores without stratification. The matching procedure yielded an average of 66 matched pairs, and the poorly matched pairs were restricted to an identical sample size in each simulation. Matched + Corrected tests and Poorly Matched + Corrected tests were performed after adjusting simulated noisy gray matter for variance in the propensity scores, then subtracting cases – controls and performing a single-sample t-test on the differences and then converting *t* to *Z* scores. Finally, Matched + Uncorrected difference scores were calculated from the paired unadjusted noisy gray matter values. Scatterplots depict increasing effect sizes from *d* = [0.5, 1.0] with darker blue to lighter blue points. When the noise bias was absent, all of the paired match methods compared were conservatively biased. A tendency for bias amplification and liberal bias emerged for Matched + Uncorrected tests as the noise bias increased, while an increasingly conservative bias was seen for the Poorly Matched + Corrected test results. When the bias was approximately 3 times larger than the difference between groups (bias = 2), these trends were amplified and a mild conservative bias was seen for the Matched + Corrected results. The boxplot summarizes the bias in the simulation results, calculated as the difference between Z_Real_ and the Z scores from the three versions of the matched pair results. This simulation provided an additional validation for the matched-pair brain morphometry method used in this study, by demonstrating how the results could be affected by match selection and adjusting the dependent variable for propensity scores.
